# Practical and emotional preparation for death: A mixed methods study investigating experiences of family carers of people with dementia

**DOI:** 10.1177/14713012211066674

**Published:** 2022-02-07

**Authors:** Emily Fisher, Sophie Crawley, Elizabeth L Sampson, Claudia Cooper, Rebecca Jones, Kanthee Anantapong, Kirsten Moore

**Affiliations:** Research Department of Clinical, Educational and Health Psychology, 4919 University College London, London, UK; Marie Curie Palliative Care Research Department, Division of Psychiatry, 325312 University College London, London, UK; Marie Curie Palliative Care Research Department, Division of Psychiatry, 325312 University College London, London, UK; Department of Psychological Medicine, Royal London Hospital, East London NHS Foundation Trust; Marie Curie Palliative Care Research Department, Division of Psychiatry, 325312 University College London, London, UK; Division of Psychiatry, 325312 University College London, London, UK; Division of Psychiatry, 325312 University College London, London, UK; Marie Curie Palliative Care Research Department, Division of Psychiatry, 325312 University College London, London, UK; Department of Psychiatry, Faculty of Medicine, Prince of Songkla University, Thailand; Marie Curie Palliative Care Research Department, Division of Psychiatry, 325312 University College London, London, UK; National Ageing Research Institute, Parkville, Victoria, Australia

**Keywords:** death preparation, death preparedness, dementia, dementia carers, end of life, end of life preparation, family carers, mixed methods

## Abstract

**Background:**

When family carers are more prepared for the end of the life of a person they care for, they report improved bereavement outcomes. Few studies have explored how carers prepare for the death of a person with dementia. We aimed to explore how carers for people with all stages of dementia experience preparing for end of life care and death.

**Methods:**

This was a mixed methods cross-sectional study. Family carers of people with dementia (*n* = 150) completed a structured interview with validated scales, alongside questions about death preparedness and advance decisions. A sub-sample (*n* = 16) completed qualitative interviews exploring their experiences of planning for end of life. We fitted logistic regression models to explore associations with preparedness, and thematically analysed qualitative data.

**Results:**

We addressed practical and emotional preparation separately for 143 participants. Fifty seven percent of participants were very practically prepared for death, while only 29% were very emotionally prepared. Male carers were more likely than female carers to report being very emotionally and practically prepared. Higher engagement with healthcare professionals was associated with feeling very practically prepared; although we found that formal discussions of end of life care issues with healthcare professionals did not impact carers’ feelings of preparation. Higher levels of dementia severity and carer depression were associated with feeling very emotionally prepared. Three qualitative themes related to practical and emotional preparation were identified: (1) ambiguity and uncertainty; (2) support from the system; and (3) how death is perceived by the carer.

**Conclusions:**

While most carers felt practically prepared for death, emotional preparation was much lower. Further research is needed to understand how engagement with healthcare professionals or other forms of social or emotional support could help carers, particularly female carers, to emotionally prepare for their relative’s death.

## Introduction

An estimated 850,000 people in the UK live with dementia (Prince et al., 2014), supported by around 700,000 unpaid family and friend carers (referred to as carers throughout) who take on the majority of care responsibility (Lewis et al., 2014). Carers often carry a physical, emotional and financial burden (Chan et al, 2013; Lindauer & Harvath, 2014). Dementia is the leading cause of death in England and Wales (Office for National Statistics, 2018), but is widely under-recognised by carers as a terminal condition (Andrews et al., 2017).

Preparation for death is multifaceted and varies according to individual circumstances, but there are key practical, medical, psychosocial and spiritual features in carers of people with dementia (Durepos et al., 2018; Hebert, Prigerson et al., 2006). More specifically, preparation for death can include dealing with legal and financial affairs, understanding disease progression, having information needs met to support end of life decision-making, acceptance of loss, accessing support from family and professionals and completing religious rituals. Greater preparation has been associated with improved carer outcomes, such as lower anxiety levels (Henriksson & Arestedt, 2013) and less complicated grief after death (Hebert, Dang et al., 2006; Schulz et al., 2003; Supiano et al., 2020), characterised as disabling distress affecting daily functioning for more than six months after the death of a significant other (Prigerson et al., 2009). Carers of people with dementia are often unprepared for the death, with studies finding a lack of preparation in one to two-thirds of carers (Hovland-Scafe & Kramer, 2016; Schulz et al., 2003).

The National Institute for Health and Care Excellence (NICE) (2018) guidelines recommend supporting people with dementia and their carers in end of life preparation through advance care planning, which involves facilitating ‘early and ongoing’ discussions about planning ahead and supporting with documentation including lasting powers of attorney and preferences about medical treatment (recommendation 1.1.12). Similarly, the European Association for Palliative Care (EAPC) guidance for dementia and end of life care states that healthcare professionals should initiate ‘timely discussions’, and patient and carers’ information and support needs should be met throughout the disease course (van der Steen et al., 2014 p.202). However, there are barriers to these discussions, including avoidance or resistance from the person with dementia (Hirschman et al., 2008), lack of knowledge and confidence of carers (Dickinson et al., 2013; Livingstone et al., 2010) and reluctance of healthcare professionals to initiate discussions for fear of causing distress (Livingston et al., 2012). Despite guidelines, there is lack of clarity around which healthcare professionals are ultimately responsible for facilitating advance care planning (Moore et al., 2019; Saini et al., 2016).

There has been little empirical research examining preparation for end of life amongst carers of people with dementia. The few studies published have explored death preparation retrospectively after death (Hebert, Dang, et al., 2006; Hovland & Kramer, 2019; Schulz et al., 2003), or in advanced stages of disease (Schulz et al., 2015) and most have been conducted in the USA (Hebert, Dang, et al., 2006; Hovland & Kramer, 2019; Schulz et al., 2015; Schulz et al., 2003). One study identified factors associated with death preparedness reported after death, and found that Black carers, carers with less education and income and more depressive symptoms were less likely to feel prepared, while those caring for someone with greater pain prior to death were more likely to feel prepared (Hebert, Dang, et al., 2006).

We aimed to extend the current literature by combining qualitative and quantitative methods to explore carer preparation for death while still caring for someone with dementia. We recruited carers in a different context – the UK, and at all stages of disease progression. Our research questions included:

1. Do carers feel prepared for end of life?
2. How do carers prepare for end of life?
3. What are the characteristics associated with preparation for death in carers?

## Methods

### Study design

This was a mixed methods cross-sectional study, interviewing family carers of people with dementia in England and Wales. We used data from the Experiencing Loss and Planning Ahead Study (ELPAS), which had a primary aim of examining the relationship between anticipatory grief and death preparedness in family carers of people with dementia (Moore et al., 2020).

### Eligibility

Inclusion criteria comprised: family or friend carers of a person with a formal diagnosis of any dementia providing practical, emotional, supervisory or social support. Participants did not have to be primary carers, and the person with dementia could be living at home or in residential care. Carers were aged 18 years or over and living in England or Wales. We excluded carers who could not communicate in English or who did not have capacity to give informed consent.

### Recruitment

Carers were recruited via the Join Dementia Research Web site, memory clinics, community mental health services and Admiral Nursing services in nine National Health Service (NHS) Trusts, Admiral Nursing services run by Royal British Legion and promotion by Alzheimer’s Society. Carers who indicated their interest were sent a Participant Information Sheet, and after three days were re-contacted to arrange a date and time for the interview if they agreed to participate.

### Data collection

All carers took part in a structured interview for quantitative analysis, and a sub-sample took part in a semi-structured interview for qualitative analysis. The structured interview and qualitative interview topic guide were informed by the literature, an academic steering group and a panel of five carers, and we trialled the interview schedule on two carers as a pilot. Written consent was given immediately before the interviews.

Validated measures were used in the structured interview, including measures of anxiety and depression (Hospital Anxiety and Depression Scale [HADS]; Zigmond & Snaith, 1983), grief (Marwit–Meuser Carer Grief Inventory [MMCGI] – short version; Marwit & Meuser, 2005), dementia knowledge (Dementia Knowledge Assessment Scale [DKAS]; Annear et al., 2017), dementia severity (Clinical Dementia Rating [CDR]; Morris, 1993) and health literacy and relationships with healthcare professionals (Health Literacy Questionnaire [HLQ]; Osborne et al., 2013). There were also questions about end of life preparation such as if the carer had discussed advance decisions with healthcare professionals, and if formal end of life care documents were in place.

To elicit a carer’s feeling of preparation we used a single question previously used in studies with family carers (Hebert, Dang, et al., 2006; Schulz et al., 2015): ‘If your loved one were to die soon, how prepared would you be for his/her death?’. Responses comprised: not at all; somewhat; very; and not sure and were dichotomised for analysis into very prepared and not very prepared (somewhat/not at all/not sure). After completing the first seven structured interviews, we revised the question on death preparedness as we found that participants struggled to respond to the single question. After asking for clarification, we found that participants could more readily respond if we distinguished between emotional and practical preparation. For the remaining interviews, we split the question into emotional and practical preparation, and added the ‘not sure’ response for those who still struggled to respond.

Interviewers recorded quantitative data from 150 structured interviews in the case report form. Carers often wanted to tell us more information beyond the structured questions, so we captured this through taking additional interview notes, including direct quotations (referred to as open notes from structured interviews throughout). The interviewer typed up these notes within 24 h of the interview taking place. The open notes from structured interviews were used in the qualitative analysis, as they contained rich data regarding personal experiences of preparing for end of life.

A purposive sub-sample of 16 took part in a qualitative interview and were selected to obtain a mix of relationship types, sex and dementia severity. This took place immediately after the structured interview or at a second date within four weeks of the initial interview if the time was not convenient for the carer or if they felt tired from the first interview. The qualitative interview explored carers’ understanding of dementia progression, the support and information received from healthcare professionals about future and end of life care needs, the timing of information about end of life, and any unmet information and support needs in relation to end of life preparation. With participants’ permission, qualitative interviews were audio-recorded on an encrypted digital recorder. Qualitative interviews were transcribed verbatim and quality checked by another researcher.

### Qualitative analysis

Transcripts from 16 qualitative interviews and open notes from 150 structured interviews were uploaded into NVivo 11 software for coding and thematic analysis (Braun & Clarke, 2006).

Three researchers with backgrounds in psychology and gerontology (EF, SC, KM) coded qualitative interview transcripts and open notes from the structured interviews. To increase trustworthiness we initially coded the same transcript and three sets of open notes from structured interviews, then compared the coding process to ensure consistency of approach. We coded the remaining data separately and met regularly to reflect on the data and share the themes we had identified, reaching theme saturation within this process. EF refined the themes to those reported in this paper, supported through regular supervisions and iterative discussion with a gerontologist and research psychiatrist (KM, KA).

We used a combined inductive and deductive approach for theme development (Fereday & Muir-Cochrane, 2006). We identified most themes inductively. These were semantic themes, relating to what the carers said explicitly and were identified using a critical realist approach to reflect the carers’ reality and explore their perceived value of preparation for death and end of life. We also identified themes deductively based on theoretical frameworks relating to carer preparation for death by Durepos et al. (2018) and Hebert, Prigerson et al. (2006). These themes were latent; going beyond the semantic level to interpret underlying concepts, patterns and meaning of the data (Braun & Clarke, 2006). Using these frameworks, we were able to identify elements of preparation and emotional domains, which carers were not able to articulate or cognisant of.

### Statistical analysis

We used summary statistics to describe the demographic and clinical characteristics of the participants. We first carried out univariable logistic analyses to explore unadjusted associations to explore associations between each outcome (practical and emotional preparedness for death; very prepared vs. not very prepared) with 13 explanatory variables including carer sex, relationship to the person with dementia, dementia severity and carer grief. These variables were identified from the literature and the results of the qualitative study, and their justification for inclusion is described in Table 1. Data from the first seven carers’ structured interviews were not included in the regression analyses because separate data on practical and emotional preparedness was not collected for these participants (see data collection).

**Table 1:** Summary of variables used in regression analyses.

Variable	Description	Justification
Age of person with dementia	Scaled per 10-year increase	Increasing age may relate to higher levels of preparation (Pinquart & Sörensen, 2002)
Sex of carer	Female, Male	Female carers may be less prepared due to lower reported lower levels of emotional well-being (Hooyman & Gonyea, 1999; Mackinnon, 2009)
Relationship to person with dementia	Adult child, partner/spouse, other	Intergenerational or relationship factors may impact preparation (Davies et al., 2014)
Residence of person with dementia	In residential care, not in residential care	To explore previously unexamined differences in preparation across disease stages. Residential care admission was expected to increase preparation
Dementia severity; clinical dementia rating (CDR) (Hughes et al., 1982;^a^ Morris, 1993)	Structured interview covering six cognitive and functional domains affected by dementia; 5-point Likert response; scoring: 0.5 very mild, 1 mild, 2 moderate, 3 severe	To explore previously unexamined differences in preparation across disease stages. Increasing severity was expected to increase preparation
Dementia knowledge; dementia knowledge assessment scale (DKAS) (Annear et al., 2017; Toye et al. 2014)	25-item response scale with four subscales; 5-point Likert response; scoring: 0–50; higher scores indicate greater knowledge	Carer understanding of progression is related to improved patient outcomes at death and may relate to death preparation (van der Steen et al., 2013)
Carer grief; Marwit-Meuser Carer grief inventory (MMCGI) – short version (Marwit & Meuser, 2005)^a^	18-item response scale with three domains; 5-point Likert response; scoring: 0–90; higher scores indicate higher levels of grief	To explore the relationship between anticipatory grief and death preparedness (Moore et al. 2020)
Carer depression; hospital anxiety and depression scale (HADS) (Zigmond & Snaith, 1983)^a^	14-item scale with two subscales; 4-point Likert response; scoring: 0–21 per subscale; higher scores indicate higher levels of depression	Depressive symptoms may be related to lack of preparation (Hebert, Dang, et al., 2006)
Carer anxiety; hospital anxiety and depression scale (HADS) (Zigmond & Snaith, 1983)^a^	14-item scale with two subscales; 4-point Likert response; scoring: 0–21 per subscale. Higher scores indicate higher levels of anxiety	Anxiety may be related to lack of preparation (Henriksson & Arestedt, 2013; McLeod-Sordjan, 2014)
Engagement with healthcare professionals; health literacy Questionnaire (HLQ) (Osborne et al., 2013)^a^	Subscale from structured interview with nine subscales; 5-point Likert response; scoring: 0–5. Higher scores indicate greater ability to engage	Healthcare professionals are expected to provide support in end of life planning (NICE, 2018) to increase preparation
Ability to navigate the healthcare system; HLQ (Osborne et al., 2013)^a^	Subscale from structured interview with nine subscales; 5-point Likert response; scoring: 0–5. Higher scores indicate greater ability to navigate	Carers expressed difficulty navigating the system in interviews; being able to navigate the system was expected to increase preparation
Discussed advance decisions and end of life with healthcare professionals	Taken place, not taken place/cannot remember	End of life discussions are a key feature of advance care planning (NICE, 2018) and were expected to increase preparation
Formal end of life care documents	In place, not in place/not sure	Formal end of life care documents discussions are a key feature of advance care planning (NICE, 2018) and were expected to increase preparation

^a^Psychometric properties of this measure can be found in the asterisked reference

We then explored independent associations using multivariable logistic analyses. For each outcome, variables from the univariable analyses with results under the threshold of *p* < 0.20 were entered into a multivariable logistic regression model simultaneously. We ensured that both models were sufficiently powered by calculating 10 participants per predictor divided by the proportion of positive cases – in this instance, carers who reported feeling very prepared (Peduzzi et al., 1996). We then carried out a stepwise backward elimination with a threshold for retention of *p* <0.05. Collinearity between covariates in multivariable models was assessed using pairwise association tests; if two covariates had collinearity of r≥0.7, one was excluded (Dormann et al., 2013). We used Stata 15.0 software, and statistical analyses were supported by a statistician (RJ).

### Mixed method approach

We first analysed quantitative data descriptively. We then analysed qualitative data with a good descriptive understanding of the sample, exploring if carers felt prepared for end of life, and how they prepared. Finally, we carried out regression analyses to explore associations with preparedness. The qualitative and quantitative results are presented separately and integrated for interpretation in the discussion.

Our mixed method approach brings together broad themes across interviews as well as specific quantifiable information from the case report form structured interview. The aim of the qualitative analysis was to identify themes in the carers’ experiences; therefore, we did not report the number of carers whose data contributed to each theme (Gilgun, 2014). Furthermore, due to the unstructured nature of qualitative data, not all topics were discussed with all participants.

## Results

### Participant characteristics

Structured interviews took place with 150 carers. This sample represents 62% of the 242 of carers who were referred to or expressed interest in the study. The number of caregivers approached by was not recorded health services, so we are unable to define the response rate. Further information about the sample is available in Moore et al. (2020). Tables 2 and 3 show the sample characteristics. One hundred and sixteen participants were female (77%), 34 were male (23%) and most were caring for a parent (*n* = 72, 48%) or spouse/partner (*n* = 70, 47%). A sub-sample of 16 carers also took part in a qualitative interview who were mostly female (*n* = 9, 56%), White British (*n* = 13, 81%), caring for a parent (*n* = 8, 50%) or spouse/partner (*n* = 5, 31%), across all stages of dementia (mild: *n* = 3, 19%; moderate: *n* = 6, 38%; severe: *n* = 7, 44%).

**Table 2.** Carer sociodemographic and clinical characteristics.

Variable	Total (*N* = 150)
*Sociodemographic characteristics*	
Age (years) – mean (SD)	63.0 (12.1)
Sex	
Female	116 (77%)
Male	34 (23%)
Ethnicity	
White British	131 (87%)
Other	19 (13%)
Relationship to person with dementia	
Adult child	72 (48%)
Spouse/partner	70 (47%)
Other	8 (5%)
Work status	
In work	52 (35%)
Not in work/retired	98 (65%)
*Validated outcome scales*	
Dementia knowledge (DKAS) – mean (SD)	34.8 (7.0)
Carer grief (MMCGI) – mean (SD)	57.6 (12.8)
Depression (HADS) – median (IQR)	5.0 (2.0–8.0)
Anxiety (HADS) – median (IQR)	8.0 (5.0–11.0)
Engagement with healthcare professionals (HLQ) – median (IQR)	3.6 (3.0–4.0)
Navigating the healthcare system (HLQ) – mean (SD)	3.4 (0.7)
*Questions about advanced decisions*	
Perceived practical preparedness	
Very	82 (57%)
Somewhat	38 (27%)
Not at all	12 (8%)
Not sure/declined to answer	11 (8%)
Perceived emotional preparedness	
Very	41 (29%)
Somewhat	56 (39%)
Not at all	24 (17%)
Not sure/declined to answer	22 (15%)
Discussed advance decisions with HCPs	
Yes	64 (43%)
No/can’t remember	86 (57%)
Formal EOLC documents in place	
Yes	63 (42%)
No/not sure	87 (58%)
Discussed EOLC wishes with person with dementia	
Yes	87 (58%)
No	63 (42%)

All statistics are counts (n) and percentages (%) unless otherwise specified. There were 7 (5%) missing observations for perceived emotional and practical preparedness. DKAS, Dementia Knowledge Assessment Scale; EOLC, End of life care; HADS, Hospital Anxiety and Depression Scale; HCP, Healthcare professional; HLQ, Health Literacy Questionnaire; MMCGI, Marwit-Meuser Carer Grief Inventory. Scale scoring in Table 1.

**Table 3.** Person with dementia sociodemographic and clinical characteristics.

Variable	Total (*N* = 150)
Age (years) – SD (range)	80.3 (9.7, 45–100)
Sex	
Female	82 (55%)
Male	68 (45%)
Age at diagnosis	
≥65	127 (85%)
<65	23 (15%)
Dementia subtype	
Alzheimer’s disease	68 (45%)
Mixed Alzheimer’s disease/Vascular dementia	21 (14%)
Vascular dementia	18 (12%)
Other	31 (21%)
Unknown	12 (8%)
Current residence	
At home	109 (73%)
Residential/nursing care/assisted living	41 (27%)
Dementia severity (CDR)	
Very mild/mild	38 (25%)
Moderate	64 (43%)
Severe	48 (32%)

All statistics are counts (n) and percentages (%) unless otherwise specified. CDR, Clinical Dementia Rating.

### Qualitative findings

Three themes were identified from the qualitative interview transcripts (*n*=16) and open notes from structured interviews (*n* = 150): (1) ‘Ambiguity and uncertainty’ with sub-themes ‘Unsure how to prepare for death’ and ‘Uncertainty in dementia progression; (2) ‘Support from the system’ with sub-themes ‘Information and discussions about end of life’ and Emotional support from professionals’; and (3) ‘How death is perceived’ with sub-themes ‘Relief in death’ and ‘Anticipating and accepting death’. The first two main themes related to both practical and emotional preparation, whilst ‘how death is perceived’ related specifically to emotional preparation.

Two sub-themes were identified based on theoretical frameworks relating to carer preparation for death (Durepos et al., 2018; Hebert, Prigerson et al., 2006). For the sub-theme ‘Unsure how to prepare for death’, we identified key elements of death preparation from these frameworks and found that many carers’ accounts highlighted there were elements missing from their understanding of death preparation, such as advance care planning or understanding end of life care wishes. For the sub-theme ‘Anticipating and accepting death’, whilst many carers did not explicitly state that they had accepted death, many carer accounts of advanced age or comorbidities were consistent with the concept of acceptance. The remaining sub-themes were identified inductively.

All quotes are attributed to a participant ID along with the source: qualitative transcript (QT) or open notes from structured interview (SI). Since a wider range of experiences were elicited from the 150 structured interviews, more quotes are sourced from the open notes from structured interviews.

### Theme 1: ambiguity and uncertainty

Many carers were unsure how to prepare for the death, and the unclear progression of dementia was a barrier to planning.

### Unsure how to prepare for death

Many carers expressed difficulty in judging their level of emotional preparation and anticipating their emotional reaction at death. Some questioned whether it was possible to be emotionally prepared.

“Well how prepared can you be? You think you are, and then it happens and then all of a sudden you’re flooded with a load of emotions that you didn’t think were there; that you don’t know how to handle,” ID82-QT (male, caring for mother with severe dementia)

Many carers struggled to come to terms with the situation and prepare emotionally for death because of their caring requirements. They described ‘putting emotions on the back-burner’ (ID 147-QT), ‘being absorbed by the day-to-day challenges’ (ID 96-SI) and ‘not having time to think’ (IDs 128-SI, 23-SI, 43-SI and 65-SI).

When carers were asked specifically about end of life care wishes of the person with dementia, some discussed preferences for place of care or death and do not attempt resuscitation orders (DNAR). Some explicitly stated they had a poor understanding of planning for end of life care. Instead many discussed after death issues, such as funerals, burial wishes and wills.

### Uncertainty in dementia progression

Carers described the uncertain prognosis as a barrier to planning, and were unsure how, or if it were possible, to plan for different possible contingencies.

“I have thought about [writing an advance care plan] but it is just impossible to do. There are so many ifs, you can’t specify all the conditions which might come up. It might be easier to do in cancer where the prognosis might be clearer. I don’t think anything is as difficult to deal with as dementia.” ID124-SI (male, caring for wife with mild dementia)

There was a perception that setting a lasting power of attorney was too soon, or that the time had not come for thinking about end of life, particularly where the person with dementia was in the mild stages. Due to this uncertainty, some carers took the approach of taking each day as it comes.

“It depends entirely on the progression of the problem as to how you’re going to tackle it, so I don’t think you can do anything until it raises its head really.” ID147-SI (female, caring for mother with moderate dementia)

### Theme 2: support from the system

Some carers expressed difficulty in navigating the health and social care system, whereas others were confident in accessing support due to previous work or caring experience or having friends or family to help them. Carers described varying levels of practical and emotional support from healthcare professionals.

### Information and discussions about end of life

Carers reported that end of life had simply not been raised as a topic, or carers had to raise it themselves. Some carers discussed not wanting these discussions, as they did not want to think ahead or think about death. Others wanted to have had the information earlier in the process, for example soon after diagnosis, during a routine check-up at the General Practitioner or memory clinic, or when the person with dementia moved into a care home.

Some carers did have end of life discussions and put documentation, for example a DNAR, in place when the person with dementia moved into a care home.

“Once she was in a care home…they really have those discussions with you… They asked me to do the do not resuscitate…So we did that via the home. That was much easier than having to think about it myself.” ID23-SI (female, caring for mother with severe dementia)

In some cases where discussions had taken place, their nature was not always sensitive or supportive in nature. One carer called DNAR a ‘tick box’ (ID94-SI) exercise and others described conversations about DNAR during hospital admissions as ‘half a corridor conversation’ (ID31-SI) or ‘blunt and with no real explanation’ (ID82-SI).

### Emotional support from professionals

Some carers did not talk to healthcare professionals, such as their GP, about the emotional nature of their caring role; either they felt that professionals were not receptive to these discussions, or they did not want to ‘burden’ professionals (ID 101-SI). Other carers described accessing formal mental health counselling, either through the NHS or privately, but it rarely related to coming to terms with the terminal diagnosis, and instead focussed on the day-to-day nature of caring and coping strategies. The following carer identified their need for counselling and found six sessions helpful to develop practical solutions:

“I did six weeks of therapy… ‘this is my problem, my head is about to explode, I’m going to go and see a therapist and talk about what’s going on and find some practical solutions’.” ID89-QT (female, caring for father with severe dementia)

Some carers described how counselling helped them to accept the situation and come to terms with their losses. One accessed counselling through a carer support charity, which took her through the five stages of bereavement. Another received support from an Admiral Nurse and trained bereavement counsellor, with whom they had recently discussed the dying process.

### Theme 3: How death is perceived

Carers generally felt prepared for the death when they perceived that the person with dementia was experiencing poor quality of life, or where they expected death due to the age of the person with dementia or their previous illnesses.

### Relief in death

Some carers felt ready for the person with dementia to die and felt it would be a relief or ‘liberation’ (ID44-SI), due to the burden and strain they experienced. Relief was often attributed to the low quality of life of the person with dementia. However, many carers expressed guilt at feeling this way.

“Part of me thinks it will be a relief and I feel awful for saying that, but you know 10 years ago if my dad could see how he is behaving now I don’t think he would like it. I feel like it will be a very sad relief because it’s like… somebody else has been put into his body.” ID19-QT (female, caring for father with moderate dementia)

### Anticipating and accepting death

Where death was expected due to old age or comorbidities, carers were more able to reflect on this, accept that death was near and prepare better emotionally. Carers discussed feeling prepared where their spouse was much older and they had expected them to die first, and carers of the oldest old (80+) discussed them having lived a long life.

“I suppose there’s also that realisation that actually the end is very imminent and of course you recognise that my dad’s in his eighties, so there’s always that kind of closeness to… the end of his life and that’s just by definition, by nature of his age”. ID9-QT (male, caring for father with severe dementia)

### Regression analyses

Table 4 shows the results from univariable analyses, using complete data from 143 structured interviews (excluding the first seven interviews). Sex had the strongest unadjusted association with both practical and emotional preparedness. The odds of female carers feeling very prepared was around one third the odds of male carers in both practical preparation (OR 0.30; 95% CI 0.12 to 0.74; *p* = 0.009) and emotional preparation (OR 0.35; 95% CI 0.15 to 0.79; *p* = 0.011). This association was retained in both adjusted models, indicating an independent association between sex and emotional and practical preparedness for death.

**Table 4.** Unadjusted associations with practical and emotional preparedness for death (*n* = 143).

	Practical preparedness			Emotional preparedness		
	Very prepared % (n)	OR (95% CI)	*p*-value	Very prepared % (n)	OR (95% CI)	*p*-value
Age of person with dementia (per 10-year increase)		1.35 (0.94–1.95)	0.109		1.07 (0.72–1.58)	0.750
Sex of carer						
Male	78% (25/32)	1 (ref)		47% (15/32)	1 (ref)	
Female	51% (57/111)	0.30 (0.12–0.74)	0.009	23% (26/111)	0.35 (0.15–0.79)	0.011
Relationship to person with dementia						
Spouse/partner	55% (36/66)	1 (ref)	0.725	31% (21/67)	1 (ref)	0.698
Adult child	61% (43/71)	1.28 (0.65–2.52)		27% (19/70)	0.82 (0.39–1.71)	
Other	50% (3/6)	0.83 (0.16–4.44)		17% (1/6)	0.44 (0.05–3.99)	
Residence of person with dementia						
At home	55% (57/104)	1 (ref)		28% (29/104)	1 (ref)	
Residential care	64% (25/39)	1.47 (0.69–3.15)	0.318	31% (12/39)	1.15 (0.51–2.57)	0.734
Dementia severity (CDR)						
Very mild/mild	51% (19/37)	1 (ref)	0.671	38% (14/37)	1 (ref)	0.057
Moderate	58% (35/60)	1.33 (0.58–3.02)		18% (11/61)	0.36 (0.14–0.92)	
Severe	61% (28/46)	1.47 (0.61–3.54)		36% (16/45)	0.91 (0.37–2.23)	
Dementia knowledge (DKAS)		1.01 (0.97–1.06)	0.573		1.00 (0.95–1.05)	0.974
Carer grief (MMCGI)		0.97 (0.94–1.00)	0.027		1.01 (0.98–1.04)	0.366
Depression (HADS)		0.93 (0.85–1.01)	0.082		1.08 (0.98–1.18)	0.114
Anxiety (HADS)		0.92 (0.84–0.99)	0.036		0.98 (0.90–1.07)	0.703
Engagement with HCPs (HLQ)		1.65 (1.09–2.50)	0.018		1.07 (0.69–1.66)	0.760
Navigating the healthcare system (HLQ)		1.69 (1.07–2.69)	0.025		1.15 (0.71–1.88)	0.568
Discussions with HCPs						
Not taken place/can’t a remember	56% (46/82)	1 (ref)		28% (23/82)	1 (ref)	
Taken place	59% (36/61)	1.13 (0.58–2.21)	0.727	30% (18/61)	1.07 (0.52–2.23)	0.849
Formal EOLC documents						
Not in place/not sure	57% (47/82)	1 (ref)		30% (25/83)	1 (ref)	-
In place	57% (35/61)	1.00 (0.51 to 1.96)	0.994	27% (16/60)	0.84 (0.40 to 1.77)	0.652

CDR, Clinical Dementia Rating; CI, Confidence Interval; DKAS, Dementia Knowledge Assessment Scale; EOLC, end of life care; HADS, Hospital Anxiety and Depression Scale; HCP, professionals; HLQ, Health Literacy Questionnaire; MMCGI, Marwit-Meuser Carer Grief Inventory; OR, Odds ratio.

Feeling very practically prepared was associated with lower levels of carer grief (OR: 0.97; 95% CI 0.94 to 1.00; *p* = 0.027) and lower levels of anxiety (OR: 0.92; 95% CI 0.84 to 0.99; *p* = 0.036) in the univariable analyses, but neither were associated with emotional preparedness. These associations with practical preparedness were not retained in the multivariable models.

Table 5 shows the results from multivariable regression analyses for practical and emotional preparedness. With practical preparation as the outcome in univariable analyses, six variables at *p* < 0.20 were added to a multivariable model (age of person with dementia, carer sex, grief, anxiety, depression and engagement with healthcare professionals). Navigating the healthcare system was excluded from the model due to high collinearity with engagement with healthcare professionals (r=0.80). The multivariable results indicate evidence of independent associations between feeling very practically prepared and carer sex and engagement with healthcare professionals. A one-point increase on the Health Literacy Questionnaire (HLQ) scale (engagement with healthcare professionals) was associated with a 61% increase in odds of feeling very practically prepared for death (AOR: 1.61; 95% CI 1.06 to 2.47; *p* = 0.027).

**Table 5.** Final multivariable models after stepwise backward elimination of associations with practical and emotional preparedness for death (*n* = 143).

	Practical preparedness			Emotional preparedness		
	Very prepared % (n)	AOR (95% CI)	*p*-value	Very prepared % (n)	AOR (95% CI)	*p*-value
Sex of carer						
Male	78% (25/32)	1 (ref)		47% (15/32)	1 (ref)	
Female	51% (57/111)	0.31 (0.12–0.78)	0.013	23% (26/111)	0.34 (0.14–0.81)	0.015
Engagement with HCPs (HLQ)		1.61 (1.06–2.47)	0.027			
Dementia severity						
Very mild/mild				38% (14/37)	1 (ref)	0.049
Moderate				18% (11/61)	0.33 (0.12–0.90)	
Severe				36% (16/45)	0.93 (0.36–2.39)	
Depression (HADS)					1.12 (1.01–1.23)	0.028

AOR, Adjusted Odds Ratio; HCPs, Healthcare professionals; HADS, Hospital Anxiety and Depression Scale; CI, Confidence Interval; HLQ, Health Literacy Questionnaire.

**Figure 1.**
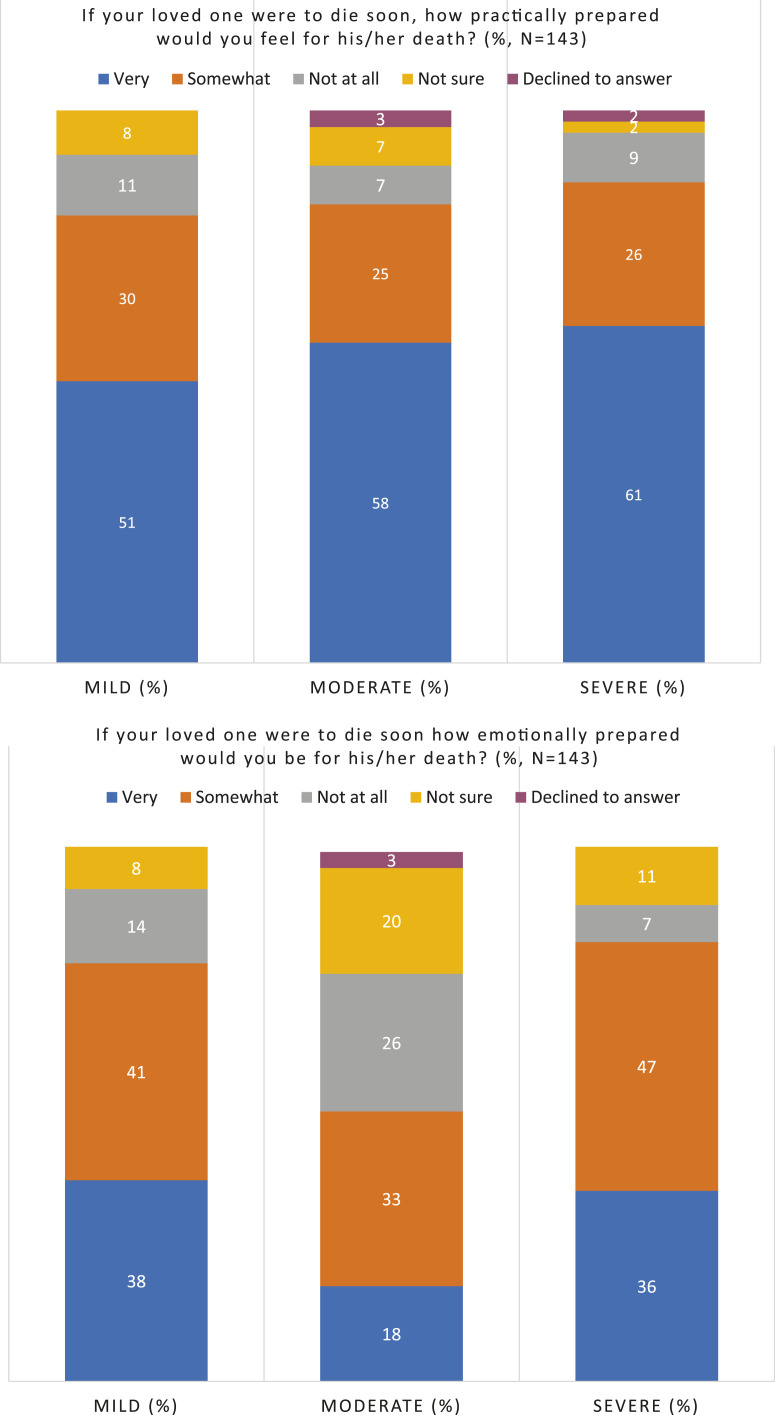
Emotional and practical preparation for end of life by dementia severity.

With emotional preparation as the outcome, three variables at *p* < 0.20 in the univariable analyses were added to a multivariable model (carer sex, carer depression and dementia severity). All three variables were retained in the model at *p* < 0 .05, indicating evidence of an association with emotional preparedness for death. A one-point increase on the Hospital Anxiety and Depression Scale (HADS) was associated with a 12% increase in odds of feeling very emotionally prepared for death (AOR: 1.12; 95% CI 1.01 to 1.23; *p* = 0.028). We found that carers of people with moderate dementia were the least emotionally prepared. Compared to carers of people with mild dementia, they had one third the odds of feeling very emotionally prepared for death (AOR: 0.33; 95% CI 0.12 to 0.90; *p* = 0.049). Carers of people with severe dementia had higher odds of emotional preparation than carers of those with moderate dementia (AOR: 2.79; 95% CI 1.08–7.20; *p* = 0.049; data not shown). Figure 1 illustrates the relationship between dementia severity and practical and emotional preparation.

## Discussion

This study builds substantially on the existing small body of knowledge relating to dementia carers’ preparation for end of life. Using both qualitative and quantitative data helps to draw a richer picture of how carers prepare for end of life and the factors influencing preparation. This is important given the projected 270% increase in people dying with dementia between 2014 and 2040 (Etkind et al., 2017).

Across all stages of dementia, we found that at least 39% of carers were not fully practically prepared and at least 62% were not fully emotionally prepared. There were high levels of uncertainty in how to prepare for the death, carers reported difficulties in accessing support in preparation from healthcare professionals, and female carers were much less likely than male carers to report feeling prepared.

### Do carers feel prepared for end of life and how do they prepare?

Carers found it very difficult to know how to prepare emotionally, and more carers felt practically prepared for death compared to emotionally prepared. There is a need to support carers to prepare emotionally, as improved preparation has been associated with less complicated grief after death (Hebert, Dang, et al., 2006; Schulz et al., 2003). Supiano et al. (2020) found that emotional aspects of preparation were associated with improved post-bereavement and grief outcomes, and the findings will inform a pre-bereavement therapy programme.

Carers seemed confident with aspects of practical preparation, especially relating to after bereavement, such as wills and funeral planning, but it was unclear if carers were aware of or understood aspects of medical preparation and types of medical treatment to be refused. Few discussed decisions around nutrition and hydration, antibiotic treatment and hospitalisation. Some carers were reluctant to think ahead to such decisions. This may have been due to uncertainty about the future or could have reflected denial about the progression of dementia. It was difficult to establish which factor had the greatest impact. Whilst not all carers will be faced with such decisions, previous studies have shown a lack of understanding of these issues can lead to uncertainty and conflict between decision makers, including family and medical staff (Lamahewa et al., 2017; Lawrence et al., 2011). Furthermore, carers’ understanding of disease progression has been found to be associated with improved patient comfort when dying (van der Steen et al., 2013).

### What are the characteristics associated with preparation for death in carers?

We found that carers with greater engagement with healthcare professionals were more likely to feel practically prepared. This probably relates to carers’ relationships with healthcare professionals throughout the disease course, their ability to have regular meaningful conversations and seek out answers to questions (Osborne et al., 2013). Despite this, many carers did not report having end of life discussions with healthcare professionals and many described receiving variable support, both in their day-to-day caring role and specifically in end of life planning. Care home admission was often a key point within the disease progression where protocols were put in place to facilitate end of life planning. However, with increasing dementia severity a key predictor of care home admission (Gaugler et al., 2009) intervention at this stage may be too late to involve the person with dementia in discussions.

Carers of those with mild dementia were more emotionally prepared for end of life than those caring for someone with moderate dementia. There are a number of possible explanations for this surprising finding. Carers of someone with mild dementia may have been less likely to have thought ahead to end of life, as reflected in the sub-theme ‘uncertainty in dementia progression’ and taking each day as it comes. So their self-reported feelings of preparation may not reflect their objective level of preparedness. Alternatively, due to the recency of the diagnosis, death may feel more salient to carers of those with mild dementia, so they may have recently confronted the emotional aspects of ageing and mortality. For those caring for someone with moderate dementia, the focus may be on immediate practical concerns associated with carer burden, such as managing neuropsychiatric symptoms or providing supervision, rather than reflecting emotionally on the situation (Chiao et al., 2015). This was evident in the sub-theme ‘unsure how to prepare for death’.

There is no universal approach to the timing of information provision, especially given the uncertainty of the prognosis of the condition, which is a barrier to preparation for death (Hovland & Kramer, 2019). However, carers of people with moderate dementia were the least emotionally prepared for death, so this stage may be a suitable time for healthcare professionals to provide information, as carers may be more receptive to support. Given the increased carer burden at this stage, (Chiao et al., 2015), carers may also be more likely to seek out information and support. Those with moderate dementia, however, may no longer have capacity to take part in these discussions and arrange documents such as lasting powers of attorney (Dening et al., 2013), so there are important considerations that should be addressed in the earlier stages of the condition.

Formal documentation of end of life care wishes and discussions with healthcare professionals about end of life were not associated with either practical or emotional preparation. Whilst some carers did share examples of perceived good practice, including healthcare professionals documenting wishes at key milestones such as care home admission, many carers described issues with the nature or timing of such discussions, for example the topic was raised without consideration of the emotional impact of the discussion. This reflects previous findings that carers often have to make complex and emotionally demanding decisions on behalf of the person with dementia with little support (Dening et al., 2012; Jones et al., 2019). Our findings suggest that end of life conversations are not routinely taking place at an early stage of the disease as recommended by NICE (2018), and that information and support needs relating to end of life are not met throughout the disease course as recommended by the EAPC (van der Steen et al., 2014).

Sex had the strongest association with both practical and emotional preparation, with male carers more likely to be prepared than female carers. Previous research has found that female carers are more likely to experience a greater emotional burden than their male counterparts (Hooyman & Gonyea, 1999; Mackinnon, 2009; Stajduhar, 2003). This has been attributed to the emotional importance that female carers place on performing well in the caring role (Hagedoorn et al., 2002), as well as the task-oriented and problem-solving approach often taken by male carers, which enables less emotional involvement compared to females (McDonnell & Ryan, 2013; Robinson et al., 2014). This task-oriented approach could be a contributing factor in the higher levels of practical preparedness in male carers. It is also possible that male carers report higher levels of preparedness in order to reflect characteristics perceived as desirable such as self-efficacy and stoicism, and to avoid appearing emotionally vulnerable (McDonnell & Ryan, 2013; Robinson et al., 2014).

We also found that carers with higher levels of depressive symptoms were more likely to feel emotionally prepared for death than those with lower levels. This finding opposes those of Hebert, Dang & Schulz (2006) where carers with more depressive symptoms were less prepared, using the self-report outcome measure of overall death preparedness (not at all vs. somewhat/very much). The authors attributed this to poor quality support at end of life being associated with depression. As our data is cross-sectional, we cannot assess causation. However a possible explanation for this unexpected finding is the depressive symptoms may precede emotional preparation. Carers who have recognised that the death of their relative may be approaching, may be emotionally prepared for that person’s death but also feel saddened by this reality.

### Strengths and limitations

This is the first mixed methods study, to our knowledge, that explores emotional and practical preparation for death in carers of people with all stages of dementia prior to bereavement. However, there are several limitations. There is currently no validated measure of preparedness for death in carers of people with dementia, so the meaning of preparation may have been interpreted in different ways (Durepos et al., 2019). The outcome could also be reflective of concepts other than death preparedness. For example, the positive association between emotional preparedness and depression could indicate that the emotional preparedness variable was measuring another concept, such as burden or resignation (Meuser & Marwit, 2001).

We had planned to ask a single, previously used question about preparation for end of life (Hebert, Dang, et al., 2006; Schulz et al., 2015), however in practice carers struggled to provide a single response to a multitude of thoughts and feelings. We therefore distinguished between emotional and practical preparation to reflect what the initial carers in the study were explaining to us. This enabled us to explore the concept in greater depth given the responses to these two aspects were very different for many carers.

The sample size of 150 is large for a qualitative study. The statistical analyses had the power to accommodate the numbers of covariates, given the number of carers who reported feeling very prepared (Peduzzi, et al., 1996). But some subgroups were small resulting in large confidence intervals, so a larger sample size would have provided more confidence in the findings. Also, due to small numbers of observations in some preparedness categories, we dichotomised the outcomes for analysis into very prepared and not very prepared. Though the range of response categories was reduced, the rich qualitative data alongside the quantitative outcomes enabled us to explore the complex nature of preparation.

Carers were recruited from a range of locations and services across England and Wales, and the sample was diverse in terms of carer sex, age, deprivation level and relationship to the person with dementia. However, as a non-random sample there is a risk of selection bias. It is possible that those who have given greater consideration to planning for death may have been more likely to volunteer for the study or be put forward by clinical teams.

We found a strong association between sex and death preparedness. We were not able to carry out thematic analysis split by sex to explore sex differences within the data, as the regression analysis followed the qualitative analysis. Future research could investigate what drives the significant disparities between male and female carers’ feelings of preparation across both domains.

## Conclusions and recommendations

Our findings highlight dementia carers’ needs for individualised and sensitive support in end of life preparation throughout the disease course. Greater emphasis may need to be placed on emotional preparation, given the difference between levels of emotional and practical preparation. However, more research is needed to understand how carers prepare emotionally for death, and how and when they can be supported to do so. This is important given the link between preparation for death and post-bereavement outcomes. Supporting carers and people with dementia to prepare for death is compatible with an approach of living well with dementia and should be a core aspect of the support carers receive.

## Appendix

### Abbreviations

CDR: Clinical Dementia Rating
DKAS: Dementia Knowledge Assessment Scale
DNAR: Do Not Attempt Resuscitation
EAPC: European Association for Palliative Care
EOLC: End of life care
HADS: Hospital Anxiety and Depression Scale
HCP: Healthcare professional
HLQ: Health Literacy Questionnaire
MMCGI: Marwit-Meuser Carer Grief Inventory
NHS: National Health Service
NICE: National Institute for Health and Care Excellence
